# Altered Expression of Chemosensory and Odorant Binding Proteins in Response to Fungal Infection in the Red Imported Fire Ant, *Solenopsis invicta*

**DOI:** 10.3389/fphys.2021.596571

**Published:** 2021-03-04

**Authors:** Zhang Wei, Almudena Ortiz-Urquiza, Nemat O. Keyhani

**Affiliations:** ^1^State Key Laboratory Breeding Base of Green Pesticide and Agricultural Bioengineering, Key Laboratory of Green Pesticide and Agricultural Bioengineering, Ministry of Education, Guizhou University, Guiyang, China; ^2^Department of Microbiology and Cell Science, University of Florida, Gainesville, FL, United States; ^3^Department of Biosciences, College of Science, Swansea University, Swansea, United Kingdom

**Keywords:** odorant binding protein, chemosensory protein, *Beauveria bassiana*, red imported fire ant, *Solenopsis invicta* Buren, fungal pathogenesis

## Abstract

Social insects have evolved acute mechanisms for sensing and mitigating the spread of microbial pathogens within their communities that include complex behaviors such as grooming and sanitation. Chemical sensing involves detection and transport of olfactory and other chemicals that are mediated by at least two distinct classes of small molecular weight soluble proteins known as chemosensory- and odorant binding proteins (CSPs and OBPs, respectively) that exist as protein families in all insects. However, to date, a systematic examination of the expression of these genes involved in olfactory and other pathways to microbial infection has yet to be reported. The red imported fire ant, *Solenopsis invicta*, is one of the most successful invasive organisms on our planet. Here, we examined the temporal gene expression profiles of a suite of *S. invicta CSPs* (*SiCSPs1-22*) and *OBPs* (*SiOBPs1-16*) in response to infection by the broad host range fungal insect pathogen, *Beauveria bassiana*. Our data show that within 24 h post-infection, i.e., before the fungus has penetrated the host cuticle, the expression of *SiCSPs* and *SiOBPs* is altered (mainly increased compared to uninfected controls), followed by suppression of *SiCSP* and select *SiOBP* expression 48 h post-infection and mixed responses at 72 h post-infection. A smaller group of *SiBOPs*, however, appeared to respond to fungal infection, with expression of *SiOBP15* consistently higher during fungal infection over the time course examined. These data indicate dynamic gene expression responses of *CSPs* and *OBPs* to fungal infection that provide clues to mechanisms that might mediate detection of microbial pathogens, triggering grooming, and nest sanitation.

## Introduction

Chemosensory and odorant binding proteins (CSPs and OBPs, respectively), represent two evolutionarily distinct protein families, that share several features (Pelosi et al., 2006, 2014; Kulmuni and Havukainen, 2013). CSPs and OBPs are small molecular weight (typically 10–18 kDa) soluble proteins capable of binding a wide range of ligands including hydrophobic and volatile compounds such as pheromones, organic and fatty acids, and other semiochemicals and environmental odors (Calvello et al., 2005; Dani et al., 2011; Pelosi et al., 2011). Transcriptome analyses continue to identify CSPs and OBPs enriched in the antennae of many insects, where they are thought to solubilize and shuttle chemical ligands to transmembrane receptors as part of olfactory and/or gustatory sensing pathways (Xue et al., 2016; Bin et al., 2017; Yuvaraj et al., 2018; Qiu et al., 2020; Yang et al., 2020). However, members of the CSP and OBP families are now recognized as functioning in a wide range of physiological processes beyond olfaction (Pelosi et al., 2018). For example, subsets of CSPs and OBPs have been found to function as reservoirs for the storage and release of semiochemicals from pheromone glands (Jacquin-Joly et al., 2001; Briand et al., 2002; Iovinella et al., 2013). Several CSPs have been shown to function in development and regeneration, and an OBP in mosquitoes has been shown to reduce host inflammation by binding leukotrienes and biogenic amines, thus blocking swelling, itching, and pain reaction in the host while feeding (Kitabayashi et al., 1998; Maleszka et al., 2007; Calvo et al., 2009). A link between CSPs and vision has been suggested by the observation of CSPs acting as carriers for β-carotene in the cotton bollworm (*Helicoverpa armigera*), and the expression of CSPs has also been linked to potential adaptive mechanisms leading to chemical insecticide resistance (Bautista et al., 2015; Zhu et al., 2016). However, to date, changes in the expression of insect CSPs and OBPs to infection by a microbial (fungal) pathogen have not been directly examined.

Red imported fire ants (*Solenopsis invicta*) are eusocial insects that live in complex societies that include communal organization, divisions of labor and reproduction, and task specialization and recruitment, all of which entail highly sophisticated social interactions and modes of communication (Wilson, 1962; Ross et al., 2007). *S. invicta* is also one of the most successful invasive species in the world, having spread from its origin in northern country-regionArgentina/southern country-regionBrazil to the United States, and from there, worldwide (Shoemaker et al., 2011). Due to the high population density of the ant nest, threats of the spread of disease-causing microbial pathogens are high. In response, these insects have evolved a number of social strategies (in addition to the innate immune system) for dealing with pathogens that include grooming and nest sanitation (Fan et al., 2012; Qiu et al., 2014). Aside from innate immune signaling (e.g., Toll-receptors), knowledge concerning mechanisms for detection and response to pathogens remains limited. The broad host range insect pathogen, *Beauveria bassiana*, is capable of parasitizing and killing *S. invicta* (Williams et al., 2003; Bextine and Thorvilson, 2004; Brinkman and Gardner, 2004). Infection begins with attachment of conidial spores to the insect integument, and subsequent growth of penetrating hyphae that enter into the insect hemolymph through the exoskeleton (Ortiz-Urquiza and Keyhani, 2013, 2015). Inside the hemolymph, the fungus undergoes a dimorphic transition, utilizing the nutrient in the hemolymph before penetrating outward to ultimately sporulate on the insect cadaver (Lewis et al., 2009; Wanchoo et al., 2009; Ortiz-Urquiza and Keyhani, 2015). Depending upon the dose, ants (and other insects) can mount successful defense strategies to counter the pathogens that include various behaviors (e.g., behavioral fever, grooming), and although infection leading to death typically progresses over a time course of 3–7 day, host (transcriptional) responses have been recorded as early as 4 h post-infection (Roy et al., 2006; Reber et al., 2011; Qiu et al., 2014; Zhang et al., 2020). These latter studies, performed in locusts, indicated potential changes in the expression of certain CSPs in response to fungal infection, although the issue was not systematically investigated.

Here, we sought to examine a time course of changes in the gene expression of 21 *S. invicta* CSPs (*SiCSPs1-4, 6-22*, note we have used the nomenclature consistent with McKenzie et al. (2014), in which there is no *SiCSP5*) and 16 *S. invicta OBPs* (*SiOBPs1-16*, no signals were seen for *SiOBP17* (Zhang et al., 2016) and this gene was not further examined) in response to *B. bassiana* infection. Expression of a large set of *SiCSPs* showed a transient stimulation 24 h post-infection, that was subsequently sharply repressed 48 h post-infection, mainly returning to uninfected levels 72 h post-infection. In contrast, changes in the expression of *SiOBPs* in response to *B. bassiana* infection were more limited, with several showing decreased expression and others increased expression during the infection course, although wave-form patterns of expression for several *SiOBPs* were noted. Of all genes examined, only *SiOBP15* showed a clear pattern of increased expression 24–72 h post-infection. These data show that CSP and OBP expression patterns can respond dynamically to pathogens during different stages of the infection process, including pre-and post-cuticle penetration, as well as during ingress and proliferation within the hemocoel.

## Materials and Methods

### Insects and Fungal Isolate

*Solenopsis invicta* colonies were collected from the field and maintained in the plastic boxes coated with talcum powder essentially as described (Fan et al., 2012). Ants were kept at 26°C with ∼70% humidity and 16:8 dark:light photoperiod. The colony was determined to be polygyne as evidenced by the presence of multiple queens and sequencing of the *Gp-9* (*OBP3*) alleles. Sucrose (300 mM solution) and freeze-dried *Galleria mellonella* larvae were fed to colonies every 2–3 day. Randomly mixed minor and major workers were used for all bioassay and RT-qPCR analysis. *B. bassiana* (ATCC 90517) was culture on potato dextrose agar (PDA) for 14–15 day at 26°C before conidia were collected in sterile 0.05% Tween-80 solution and mycelial debris were removed by filtration through sterile lens paper. Spores concentrations were calculated by direct counting using a Neubauer hemocytometer.

### Insect Bioassays

Various concentrations of *B. bassiana* conidia were prepared via dilution to 1 × 10^5^, 1 × 10^6^, 1 × 10^7^, and 1 × 10^8^ conidia/mL in sterile 0.05% Tween-80. *S. invicta* workers (20/replicate × 3 replicates/experiment) were immersed in the conidial suspensions for 15 s, and the workers were removed and placed on a dry tissue paper. Control ants were treated with 0.05% Tween-80. Controls and treated workers were kept at 26°C with ∼70% humidity and 16:8 dark:light photoperiod and placed in standard Petri dishes containing an Eppendorf tubes filled with sucrose (300 mM) solution, that was replaced every 2–3 day. Mortality was recorded twice daily and dead insects were removed and placed in tubes under 70% humidity to confirm fungal outgrowth. The entire experiment was repeated with three independent batches of fungal conidia.

### Gene Expression Analyses: RT-qPCR

Workers treated with suspensions of 1 × 10^8^ *B. bassiana* conidia/mL as above were collected for quantitative RT-PCR gene expression analyses. Controls were treated with 0.05% Tween-80. After treatment, surviving workers were collected over a time course of the infection that included 12, 24, 48, and 72 h post-inoculation. Total RNAs were extracted from the whole body of workers using TRIzol reagent (Invitrogen, Carlsbad, CA, United States), and contaminating genomic DNA was removed by digestion using TURBO DNase (Invitrogen) according to the manufacturer’s instructions. Agarose gel electrophoresis and NanoDrop 2000 spectrophotometric analyses were performed to determine the quality and quantity of the total RNA preparations. An aliquot of 2 μg of the purified total RNA was used to construct cDNA libraries from each sample, using the High-Capacity cDNA Reverse Transcription Kit (Applied Biosystems, Foster City, CA, United States). Primers for RT-qPCR (Supplementary Table 1) were designed using Beacon Designer 8 software. The *S. invicta* elongation factor-α (EF1α) gene was used as the housekeeping (reference) gene. The amplification efficiencies of all primers were confirmed by empirical construction of standard curves and primer concentrations and annealing temperatures were determined according to the derived amplification efficiencies (Zhang et al., 2016; Wanchoo et al., 2020). For RT-qPCR experiments, the *S. invicta* cDNA libraries from various treatments were diluted 40-fold in nuclease-free ddH_2_O (double-distilled H_2_O), and 5 μl of diluted cDNA was used as the template in a 15 μL reaction volumes. Each reaction contained 1× Master Mix (Biotools, Houston, TX, United States), 5 μL template and 200 nM of each of gene specific primer pairs. All reactions were performed in triplicate. At least three independent RNA preparations for each sample were analyzed using the RT-qPCR protocol. The RT-qPCR reactions were performed using an Eco Real-Time qPCR System (Illumina, San Diego, CA, United States) with a thermo-profile of one cycle of 95°C 5 min, 95°C 2 min, then 45 cycles of 95°C 15 s, and 60/59°C 45 s, followed by a melting curve analysis from 55 to 95°C.

### Phylogenetic Analyses

Chemosensory proteins and OBPs protein sequences from the ant species *Linepithema humile* (Argentine ant), *Camponotus floridanus* (carpenter ant), *Camponotus japonicus* (Japanese carpenter ant), *Hapergnathos saltator* (Jerdon’s jumping ant), and *S. invicta* were used to build limited phylogenetic trees (Supplementary Tables 2, 3, and Supplementary Material). For both CSP and OBP sequences, amino acid multiple sequence alignments were generated with webPRANK (Löytynoja and Goldman, 2010), and the best fitting models for amino acid substitutions were estimated with MEGA7 (Kumar et al., 2016). MEGA7 chose the models LG + G and JTT + G for the CSP and OBP sequences, respectively, and these models were used to build the phylogenetic trees with RaxML, available at the CIPRES Science Gateway (Miller et al., 2010; Stamatakis, 2014). G (gamma shape parameters) and branch length were estimated, and branch support was calculated by bootstrapping. RaxML was allowed to execute 1000 rapid bootstrap inferences and halt bootstrapping automatically after a thorough maximum likelihood search (934 bootstraps). The software MEGA7 was used to draw the tree (Kumar et al., 2016).

### Data Analyses

Normalized expression data were examined by one-way ANOVAs (with *post hoc* comparisons using Bonferroni and Duncan’s test) using the IBM SPSS Statistic 20 software package (SPSS, Inc., 2011). The mean lethal time (LT_50_) and mean lethal concentration were estimated by Probit analysis.

## Results

### Phylogenetic Analyses

**FIGURE 1:**
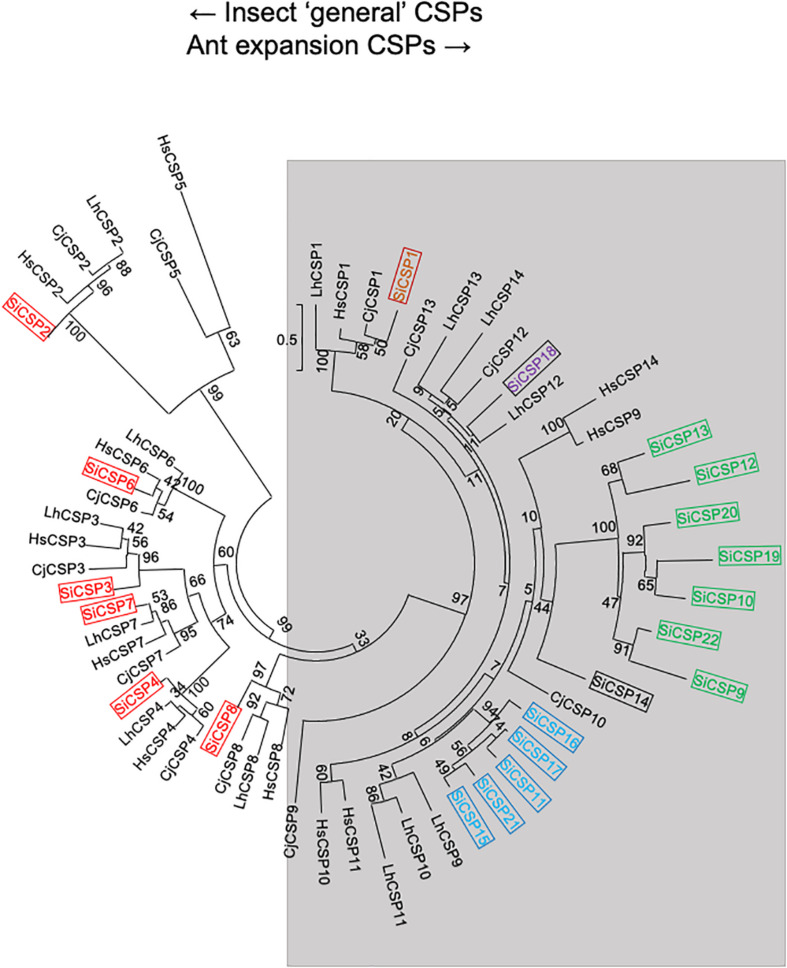
Simplified phylogenetic tree of S. invicta CSPs. Limited maximum likelihood phylogeny of S. invicta CSPs compared to the CSP repertoires found in the ant species, C. japonicus (CjCSP), H. saltator (HsCSP), and L. humile (LhCSPs) (Accession numbers given in Supplementary Table 2). Numbers at nodes indicate bootstrap values. Scale shows number of substitutions per site. The tree is midpoint-rooted in the absence of a suitable out-group. More detailed phylogenetic analyses of insect CSPs can be found in Kulmuni and Havukainen (2013), McKenzie et al. (2014).

**FIGURE 2:**
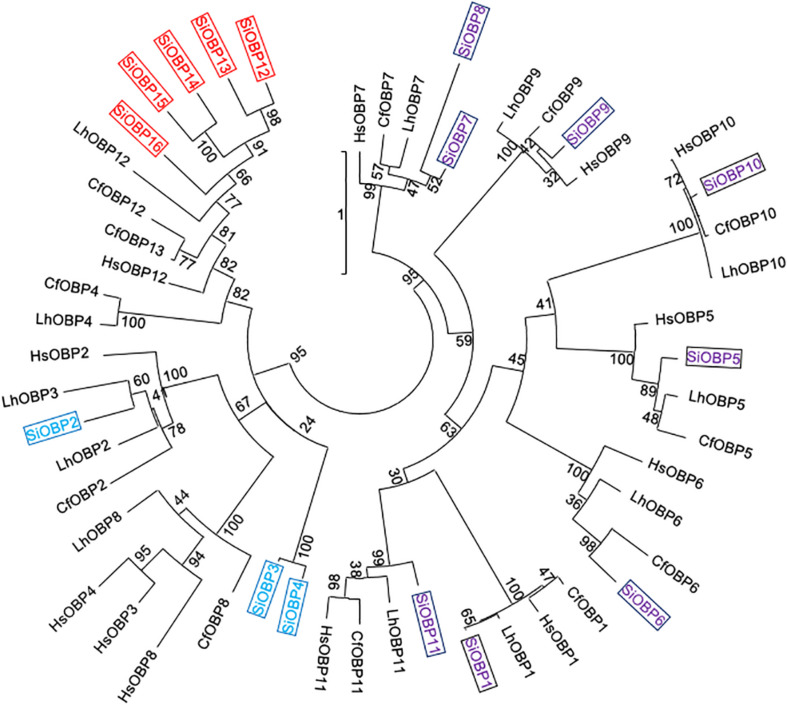
Simplified phylogenetic tree of *S. invicta* OBPs. Limited maximum likelihood phylogeny of *S. invicta* OBPs compared to the OBP repertoires found in the ant species, *C. floridanus* (CfCSP), *H. saltator* (HsCSP), and *L. humile* (LhCSPs) (Accession numbers given in Supplementary Table 3). Numbers at nodes indicate bootstrap values. Scale shows number of substitutions per site. The tree is midpoint-rooted in the absence of a suitable out-group. More detailed phylogenetic analyses of insect CSPs can be found in McKenzie et al. (2014), Xue et al. (2016), Zhang et al. (2016).

The amino acid sequences for 21 SiCSPs (SiCSP1-4 and 6-22) and 16 SiOBPs (SiCSP1-16) were used to construct limited phylogenetic trees including CSPs and OBPs from the ant species *S. invicta*, *L. humile*, *C. floridanus*, *C. japonicus*, and *H. saltator* (Figures 1, 2). As has been reported by others (Gotzek et al., 2011; Kulmuni and Havukainen, 2013; McKenzie et al., 2014), for the CSPs, these analyses revealed a division of the CSPs into ant-specific lineages and those shared with other insects. Within the ant-specific lineages, SiCSPs 1, 14 (somewhat related to the CSP gene expansion labeled in green), and 18 were dispersed in the tree, whereas two distinct fire ant CSP gene expansions (boxed in green and blue) were noted (Figure 1). These expansions consisted of SiCSPs 9, 10, 12, 13, 19, 20, and 22, in one clade (green), and SiCSPs 11, 15, 16, 17, and 21 in another (blue). SiCSPs 2, 3, 4, 6, 7, and 8 showed homology to CSPs found in order insects. Unlike CSPs, no ant specific OBPs were noted. SiOBPs 12-16 formed a *S. invicta*-specific OBP expansion (Figure 2), with the other OBPs dispersed within the tree. SiOBP3, which showed high amino acid similarity with SiOBP4 (Figure 2), is equivalent to GP-9 that has been linked to a “mini-chromosome” which also contains SiOBPs 4, 5, 9, 12, 13, and 15-16, that appears to show limited recombination and has been implicated as mediating important aspects of ant “social” behavior (Linksvayer et al., 2013; Nipitwattanaphon et al., 2013; Wang et al., 2013; Zhang et al., 2016).

### Ant Bioassays

**FIGURE 3:**
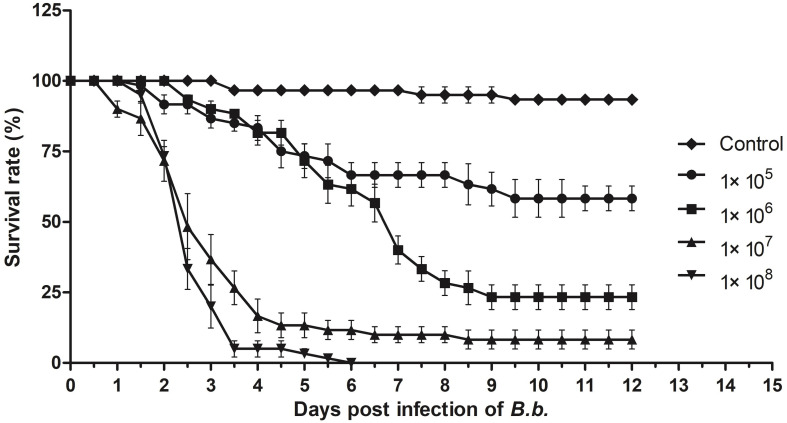
Insect bioassays. Time course of infection of *S. invicta* workers by *B. bassiana* using indicated concentration of fungal conidia as the inoculum. All experiments were performed in triplicate. Error bars = ± SD.

As a broad host range insect pathogen, *B. bassiana* is known to be able to infect ants, including *S. invicta* (Stimac et al., 1993; Oi et al., 1994), and insect bioassays indicated dose-dependent mortality of *S. invicta* after parasitism by *B. bassiana* (Figure 3). At low doses (10^5^–10^6^ conidia/mL) used in this work, infection yielded ∼25 and 60% mortality, respectively, whereas at higher doses (10^7^–10^8^ conidia/mL), almost all treated individuals eventually succumbed to the infection. The mean lethal time (LT_50_) for the latter two doses (10^7^–10^8^ conidia/ml) was calculated as being 3.2 ± 0.63 h and 2.5 ± 0.2 days, respectively. Based on these data, an approximate mean lethal dose for 50% mortality (LD_50_) was calculated to be 1.13 × 10^7^ conidia/mL.

### Expression of *CSPs* and *OBPs* in *B. bassiana* Infected and Non-infected *S. invicta* Workers

**FIGURE 4:**
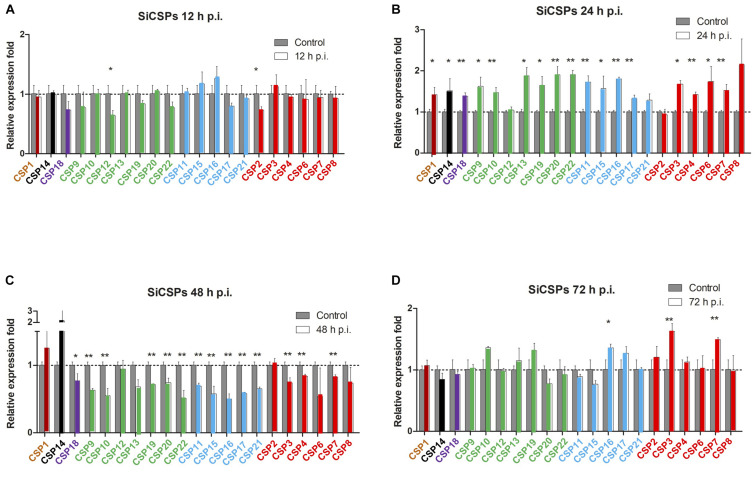
Gene expression analyses of *S. invicta* CSPs in response to *B. bassiana* infection. *S invicta* workers were infected using 1 × 10^8^ conidia/ml. Total RNA was isolated **(A)** 12 h, **(B)** 24 h, **(C)** 48 h, and **(D)** 72 h post-infection and RT-qPCR reactions performed as detailed in the “Materials and Methods” section. CSPs are grouped and color coded according to phylogenetic analyses shown in Figure 1. All experiments were performed in triplicate. Error bars = ± SD. Symbols “*” and “**” indicate statistical significances of *P* < 0.05 and *P* < 0.01, respectively.

**FIGURE 5:**
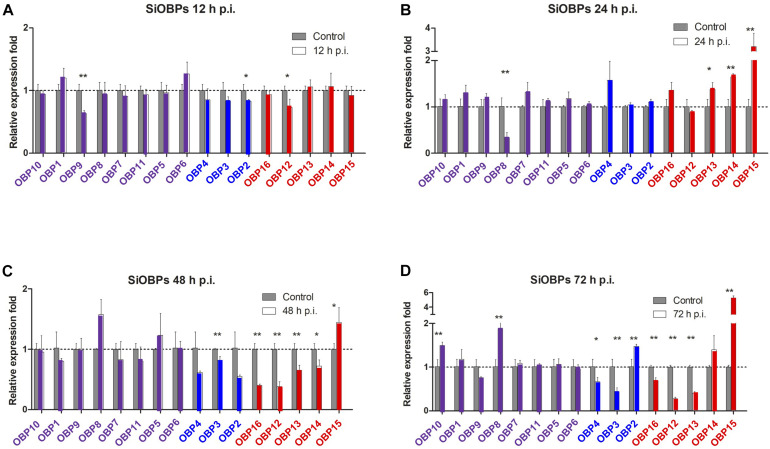
Gene expression analyses of *S. invicta* OBPs in response to *B. bassiana* infection. *S invicta* workers were infected using 1 × 10^8^ conidia/ml. Total RNA was isolated **(A)** 12 h, **(B)** 24 h, **(C)** 48 h, and **(D)** 72 h post-infection and RT-qPCR reactions performed as detailed in the “Materials and Methods” section. OBPs are grouped and color coded according to phylogenetic analyses shown in Figure 2. All experiments were performed in triplicate. Error bars = ± SD. Symbols “*” and “**”indicate statistical significances of *P* < 0.05 and *P* < 0.01, respectively.

Changes in the expression of *SiCSPs 1-4*, *6-22*, and *OBPs 1-16* were examined by RT-qPCR in *S. invicta* workers over a time course (12–72 h) of *B. bassiana* infection using 10^8^ conidia/mL as detailed in the “Materials and Methods” section (Figures 4, 5). CSPs are grouped by their phylogenetic position and color coded as in Figure 1. No sequence for *SiCSP5* was assigned as per previously reported nomenclature (McKenzie et al., 2014). During 12 h post-infection only *SiCSP13*, within the ant specific expansion and *SiCSP2*, found in the “insect general” *CSP* clade were significantly (*P* < 0.01) downregulated as compared to untreated controls. However, by 24 h post-infection, the expression levels of a large set of *SiCSPs* showed significantly increased expression in response to *B. bassiana* infection. These included *SiCSPs 1*, *14*, *18* (ant-specific), and members of the sub-clusters, *SiCSPs 13*, *19*, *20*, and *22* (in green, fire ant *CSP* gene expansion), and *SiCSPs 11*, *15*, *16*, and *17* (in blue, representing a second fire ant *CSP* expansion), as well as the “general” *SiCSPs 3*, *4*, *6*, and *7*. With the exception of *SiCSP14*, which showed a dramatic significant increase in expression 48 h post-infection, the increased expression of the other *SiCSPs* appeared transient, and was followed by a sharp decrease in expression levels. Indeed, aside from a handful of *SiCSPs*, whose expression did not vary significantly (i.e., *SiCSPs 1*, *2*, *8*, *12*, and *13*), the expression of the other *SiCSPs* was significantly decreased in infected ants compared to controls. By 72 h post-infection, *SiCSP* expression levels were similar to controls, except for *SiCSP16*, *3*, and *7*, which were significantly upregulated (Figure 4).

With respect to the *SiOBPs*, at 12 h post-infection and similar to the *SiCSPs*, significant decreases in the expression of only a handful of *SiOBPs*, namely *SiOBPs 2*, *9*, and *12* were seen (Figure 5). By 24 h post-infection, only *SiOBP8* showed significantly decreased expression in response to *B. bassiana* infection, whereas *SiOBPs 4, 13*, and *15*, (with the latter >3–4 fold) showed significant upregulation in response to infection. At 48 and 72 h post-infection, *SiOBPs 3*, *12*, *13*, and *16* showed decreased expression, whereas *SiOBP14* and *SiOBP4* showed significantly (*P* < 0.01) decreased expression at 48 and 72 h post-infection, respectively. *SiOBP15* was the only *OBP* (and gene examined) to show a consistent pattern of increased expression throughout the 24–72 h post-infection time course. Expression of *SiOBP3/gp9* gradually decreased during *B. bassiana* infection and was at ∼50% 72 h post-infection.

## Discussion

Though the functions of some CSPs and OBPs in chemical perception and potential downstream behavioral regulation have been reported, any roles during microbial pathogen infection have not been systematically studied. The data presented here suggest a number of important points. First, that *SiCSP* and *SiOBP* expression is significantly more dynamic than previously considered. Second, to the best of our knowledge, these data are the first to show systematic changes in expression of the suite of *CSP* and *OBP* genes in response to microbial infection. In addition, our data show that changes in (fire ant) *CSP* and *OBP* expression occur very early in the infection process–being seen within 24 h post-infection, a time point before the fungus has penetrated the cuticle which typically occurs 24–48 h after infection. In addition, *SiCSP* and *SiOBP* gene expression responses did not follow any clear phylogenetic patterns, i.e., more closely related *SiCSPs/SiOBPs* did not appear to follow similar gene expression responses to *B. bassiana* infection than more distantly related *SiCSPs/SiOBPs*.

Upon *B. bassiana* infection, *SiCSPs* and *SiOBPs* showed some similar temporal expression dynamics, with expression of these genes downregulated, in general, at the very initial stages of the infection (i.e., likely at the initiation of germination at 12 h post-infection) and subsequently upregulated at 24 h post-infection, i.e., once most of the conidia were probably germinated and starting the processes of breaching the cuticle. At 48 h (i.e., initial hemocoel colonization), *SiCSP* and *SiOBP* expression lowered, and only a few *SiCSPs* and *SiOBPs* were upregulated at 72 h post-infection (i.e., hemocoel colonization). Two important aspects should be noted. First, many CSPs and OBPs are not involved in antennal chemosensation and instead may function as ligand carriers in other physiological functions that can include pheromone and hormone sequestration and signaling (Dani et al., 2011). Second, our experimental design does not discriminate between any self- versus allo-grooming that may occur.

Among the *SiCSPs*, the most significant increase occurred for *SiCSP14* at a time point in which expression of most other *CSPs* was sharply decreased (i.e., 48 h post-infection). Prior studies reported high and robust expression of *SiCSP14* in the worker abdomen and antennae, respectively, while showing low expression in the head and the thorax (Wanchoo et al., 2020). These gene expression analyses showed that *SiCSP3*, *7* and *17*, which were the only *CSPs* showing a change of expression in infected ants 72 h post-infection (∼1.5-fold increase), exhibited robust and high expression in the antennae and the abdomen of workers (Wanchoo et al., 2020). Of the major fire ant antennal *SiCSPs* (*SiCSPs12, 8, 19, 11, and 1*) (Wanchoo et al., 2020), *SiCSP19* and *11* showed a significant increase in expression at 24 h post-infection, followed by a decrease at 48 h post-infection. Of the three other most-expressed antennal *SiCSPs*, *SiCSP12*, and 8 showed no change in expression over time, whereas *SiCSP1* displayed a very transitory and slightly increase in expression 24 h post-infection that dropped to uninfected levels 48 and 72 h post-infection. These results again highlight the potential for dynamic changes in CSP expression patterns to environmental stimuli (including microbial infection) that has hitherto been neglected.

In *S. invicta* worker antennae, gene expression analyses indicated high expression of *SiOBPs 1*, *2*, *5*, and *6* (Zhang et al., 2016), none of which showed dramatic responses to *B. bassiana* infection. In worker head tissues, aside from high expression of *SiBOP3*, *SiOBPs 2*, *7*, *10*, *13*, and *15* were also highly expressed, and as noted *SiOBP15* was the only gene examined whose expression was consistently increased during *B. bassiana* infection. *SiOBP15* was also not very highly expressed in worker thorax and abdomen tissues (the latter showing low expression of *SiOBPs* in general) (Zhang et al., 2016). Intriguingly, *SiOBP8*, which showed the greatest increase in response to *B. bassiana* infection after *SiOBP15*, was in general poorly expressed in worker tissues (Zhang et al., 2016).

*Solenopsis invicta OBP14* which was highly expressed in worker thorax, along with *SiOBP3*, (Zhang et al., 2016), showed an initial decrease in expression followed by a significant increase (*P* < 0.01) as compared to untreated controls 48 h post-infection. *SiOBP3* (*gp-9*) has been implicated as part of a control locus that mediates aspects of social behavior, notably mono- versus polygyny in fire ants (Gotzek and Ross, 2009). However, it is now recognized as being part of a significantly larger “mini-chromosome” that includes additional OBPs (i.e., *SiOBPs 4*, *5*, *9*, *12*, *13*, and *15-16*) and CSPs (i.e., *SiCSP7, 9, 12, 13, 14, and 22*) (Zhang et al., 2016), and which appears to be restricted in terms of recombination (i.e., linkage group 16) (Wang et al., 2013; Nipitwattanaphon et al., 2014). Thus, it is possible that this genomic region includes a range of genes involved in a network of regulation that ultimately impacts social organization. Expression of *SiOBP3*, together with *SiOBPs 12*, *13*, and *16*, appeared to respond to *B. bassiana* showing a consistent downward trend in expression levels 48 and 72 h post-infection.

A range of studies have indicated a clear role for soluble “olfactory” proteins in physiological processes beyond olfaction. Our data show that this can now be potentially expanded to include response to (microbial) pathogens. It remains to be determined whether these functions are within olfaction, e.g., recognition of the fungal pathogen–spores or other infectious propagules–on the insect surface, or downstream processes, e.g., inflammation, development/reproduction, regeneration and/or a combination of both. Within the olfaction context, it is known that ants engage in social behaviors that include grooming and sanitation (Reber et al., 2011; Qiu et al., 2014), and therefore CSPs and/or OBPs may be involved in the chemosensation of microbial pathogens on the insect surface. In addition, insect responses to pathogens can include changes in feeding and reproduction that may be mediated by hormones and other signaling molecules during the infection process. As CSPs/OBPs can act as carriers for these molecules, changes in their expression levels may reflect responses to the infectious agent.

As in most cases examined, changes in the expression of *CSPs*/*OBPs* were transient and appeared to follow a wave-like pattern, showing increased expression within 24 h post-infection, followed by decreased expression by 48 h post-infection. At 12 h post-infection, the fungal conidia have attached and germinated on the insect surface but have yet to penetrate the cuticle (Ortiz-Urquiza and Keyhani, 2013). Large scale transcriptomics have revealed similar changes in gene expression patterns during pre-penetration events in locusts infected by the insect fungal pathogen *Metarhizium acridum* (Zhang et al., 2015, 2020). These data indicate that insects can detect microbial pathogens early during infection and hence may attempt to quickly mobilize immune or other responses to the infection. In this context, signals (lipids and other compounds) on the insect surface are known to change as the fungus germinates and germ tubes begin to grow on the surface before penetration (Pedrini et al., 2010, 2013). At 24 h post-infection, fungal hyphae are in the process of breaching the cuticle and by 48 h can reach the hemolymph (Ortiz-Urquiza and Keyhani, 2016). It is during these stages that our data show high fluctuation (increase then decrease) in *SiCSP* and *SiOBP* expression levels, that may reflect the transition to elicitation of direct (innate) immune responses once the fungus has breached the cuticle. At 72 h post-infection, the fungus is likely proliferating within the hemocoel, forming free-floating hyphal bodies that are capable of evading immune surveillance (Lewis et al., 2009; Wanchoo et al., 2009). At this stage, changes in *SiCSP* and *SiOBP* expression levels appear to be limited. Overall, our results highlight that insect responses may be calibrated to infection dynamics and that the time course of the infection needs to be considered in any examination of such responses. Both suppression and induction of *SiCSPs* and *SiOBPs* were noted, with *SiOBP15* showing the most consistent (increased) response across the infection time course. These data allow for discrete functional hypotheses to be made concerning a number of *SiCSPs* and *SiOBPs* that may be implicated in microbial infection responses and future work examining several candidates within the context of (*B. bassiana*) infection is warranted.

## Data Availability Statement

The raw data supporting the conclusions of this article will be made available by the authors, without undue reservation.

## Author Contributions

NOK and ZW initiated the project and conceived and designed the study. ZW, AO-U, and NOK performed the samples collection, library constructions, RT-qPCR, data processing, bioinformatic analyses, data interpretation, wrote the manuscript, and contributed to revisions of the manuscript. All authors contributed to the article and approved the submitted version.

## Conflict of Interest

The authors declare that the research was conducted in the absence of any commercial or financial relationships that could be construed as a potential conflict of interest.

## Supplementary Material

Supplementary Table 1 List of primers used for RT-qPCR analyses.

Supplementary Table 2 CSPs accession numbers.

Supplementary Table 3 OBPs accession numbers.

Supplementary Material Amino acid sequences of CSPs and OBPs used for the phylogenetic analyses.
