# Supplementary material for: Altered Expression of Chemosensory and Odorant Binding Proteins in Response to Fungal Infection in the Red Imported Fire Ant, Solenopsis invicta
Source: Front Physiol. 2021 Mar 4;12:596571. doi: 10.3389/fphys.2021.596571 (PMC7970113; doi:10.3389/fphys.2021.596571)
Supplement: Supplementary Table 1 — List of primers used for RT-qPCR analyses. [file Table_1.DOCX]

**Supplemental Materials**

**Altered expression of chemosensory and odorant binding proteins in response to fungal infection in the red imported fire ant, *Solenopsis invicta***

Zhang Wei^1,2^, Almudena Ortiz-Urquiza^2,3^, and Nemat O. Keyhani^2*^

**Supplemental Table S1. List of primers used for quantitative RT-PCR primers**

|  | Forward | Reverse |
| --- | --- | --- |
| SiCSP1 | TGCTGGCCATTCTGGCTGT | CGAAGCTCATCGTTCGGCA |
| SiCSP2 | CAACTGAACATAGCCCTGAG | GATCTGACGTATCTCTTCGG |
| SiCSP3 | CTGCTGAAGCTTTAGAGGC | CATCCTCATACTTCTTGGCG |
| SiCSP4 | GGATGTAGCGAGACGCAGAA | TAACGCCGTCGATATGTCCC |
| SiCSP6 | CCTCCAGAATAATCGCGTTC | GATCGTATTTGGCGACGA |
| SiCSP7 | CGAGAAGGTCATCCGGTATC | GGCCATTGGGATCATACTTC |
| SiCSP8 | GATAAAGGTCCCTGTACTGC | CTCTCGAGAAATTCTGCCC |
| SiCSP9 | CTTGCAGAAGACCTCCATAG | CCAAGTATCGGGTTGGTTC |
| SiCSP10 | AAGAGTACCGTGCGCAAAAG | TCCAAGTATCGGGTTCGTTC |
| SiCSP11 | CGTAACAGCCGATGCAAAGTTC | ACGGCAGCTTGCCATTTATC |
| SiCSP12 | GGACCGTGCACACTAGAACA | AGTGCATCTTCTGCAGTTGG |
| SiCSP13 | TATCGCGTTGCTCGTTGTTG | TTCTGCGCACGGTTCTCTTT |
| SiCSP14 | ATGCGTTCTCGCGAAAGAAGAG | ACGTGCACGGTGTTATTCCC |
| SiCSP15 | TTGCACTAATGTGCGTTGCC | GTTACGCACGACGATGTTCC |
| SiCSP16 | AAAACAGCCGATGCGAAG | AGCTTGCCACTCATCAGGTT |
| SiCSP17 | AGGGGTTTTTGCAGATGCTC | GAGAGCCTGCCATTCATCAG |
| SiCSP18 | CCGCAGATGCGAAATTCT | GAAGGCTTCCCATTGTTCAG |
| SiCSP19 | AAAGGACCGTGCTCAGAAGA | GGCTTCGTTCTGTTCGTACC |
| SiCSP20 | CGTACTGATGTGCGTTCTTGG | CTTCTGTACACGGTGCTCTT |
| SiCSP21 | ATCGCCATGCGTAACAG | CCTCAGATTTTGCAACGGC |
| SiCSP22 | GCGACAGAAAGCAGTCGAT | TCCAAGTATCGGGTTGGTTC |
| SiOBP1 | TGACCGGTCTATCACGTGCT | GACGCATTTCGCCACATTGT |
| SiOBP2 | TCATTGAAAGAGTGGGCCGAAT | GCAGTCAGGCATTCCGTTAGA |
| SiOBP3 | TGAAGATACCGAACTACACAA | AGCACCTGTTTCCTTGAT |
| SiOBP4 | AGCTCTTCTCGGTTGGTGAA | GCCCAAGCTTTTGTCACATT |
| SiOBP5 | ACGCATAGATGACGGTAT | TGATAAGTGGTTGAGCAATC |
| SiOBP6 | ATATTTGCCAGTTGTCGTAAA | ATCGTTGGATTAACATCATAGAG |
| SiOBP7 | AAAGGAGAGGTAGCTGAAAGTGAC | TGCCTCCTTTTTCACAACCTGT |
| SiOBP8 | TTAAGAGGCTACAGAACATTATG | AGAGTTACCTTCCAGTCAAT |
| SiOBP9 | AGGCTGGAAACATTGAACAAAA | TATCGCATGCATTCTCCGCT |
| SiOBP10 | TTGGTCTGGTCGACGACAAG | GGAGTAGCTGCGCGTAACA |
| SiOBP11 | TTCTCGTCTGTGTCTCTG | TCTGCTCCTTCTAACTTATCA |
| SiOBP12 | TGCTGTGCGCATGTGTTTTG | ACACGCCATAAAGCAGCCAT |
| SiOBP13 | GCTGTGCGCATGTGTTTTGA | ACACGCCATAAAGCAGCCAT |
| SiOBP14 | CCCACCAGCCGAAATAATAGCT | CGCAGCCGCTTCTTTTACG |
| SiOBP15 | GCTGACTTTGTCAAACCGATGG | AATTTGGTGCATTGGTCCGC |
| SiOBP16 | AAAAGTCTCGTGCTTTGCGC | TGCAACAAAGCAACCAACT |
| SiOBP1 | TGACCGGTCTATCACGTGCT | GACGCATTTCGCCACATTGT |
| EF1-α | AAGAGAACCCGAAAGCCATT | GCCTCAACGCACATAGGTTT |

**Table S2. Accession numbers of the chemosensory proteins (CSP) used in the phylogenetic analysis shown in Fig. 1**

| Species | CSP | BLAST accession number |
| --- | --- | --- |
|  | CjCSP1 | BAS29775 |
|  | CjCSP2 | BAS29776 |
|  | CjCSP3 | BAS29777 |
|  | CjCSP4 | BAS29778 |
|  | CjCSP5 |  |
| *Camponotus japonicus* | CjCSP6 |  |
|  | CjCSP7 | BAS29779 |
|  | CjCSP8 |  |
|  | CjCSP9 |  |
|  | CjCSP10 |  |
|  | CjCSP12 | BAS29780 |
|  | CjCSP13 | BAS29781 |
|  | HsCSP1 | XP_011151735 |
|  | HsCSP2 | XP_011154714 |
|  | HsCSP3 | XP_011153546 |
|  | HsCSP4 | XP_011153548 |
|  | HsCSP5 | XP_011152650 |
| *Harpegnathos saltator* | HsCSP6 | XP_011152649 |
|  | HsCSP7 | EFN85227 |
|  | HsCSP8 | XP_011138157 |
|  | HsCSP9 | EFN81741 |
|  | HsCSP10 | XP_011137361 |
|  | HsCSP11 | XP_011137370 |
|  | HsCSP14 | XP_011143827 |
|  | SiCSP1 | XP_011160273 |
|  | SiCSP2 | EFZ11660 |
|  | SiCSP3 | XP_011163782 |
|  | SiCSP4 | XP_011163783 |
|  | SiCSP6 | XP_011166108 |
|  | SiCSP7 | XP_011172450 |
|  | SiCSP8 | EFZ17227 |
|  | SiCSP9 | XP_011164980 |
| *Solenopsis invicta* | SiCSP10 | XP_011160350 |
|  | SiCSP11 | XP_011160226 |
|  | SiCSP12 | XP_011164990 |
|  | SiCSP13 | EFZ22773 |
|  | SiCSP14 | XP_011159610 |
|  | SiCSP15 | XP_011160225 |
|  | SiCSP16 | EFZ17972 |
|  | SiCSP17 | EFZ17504 |
|  | SiCSP18 | XP_011168346 |
|  | SiCSP19 | XP_011160348 |
|  | SiCSP20 | XP_011160349 |
|  | SiCSP21 | XP_011160224 |
|  | SiCSP22 | XP_011164970 |
|  | LhCSP1 | NP_001296370 |
|  | LhCSP2 | XP_018301216 |
|  | LhCSP3 | XP_012231377 |
|  | LhCSP4 | XP_012231523 |
|  | LhCSP6 | XP_012226579 |
| *Linepithema humile* | LhCSP7 | XP_012226485 |
|  | LhCSP8 | XP_012217526 |
|  | LhCSP9 | XP_012223737 |
|  | LhCSP10 | XP_012218566 |
|  | LhCSP11 | XP_012218565 |
|  | LhCSP12 | XP_012217907 |
|  | LhCSP13 | XP_012220466 |
|  | LhCSP14 | XP_012220375 |

**Table S3. Accession numbers of the odorant binding proteins used in phylogenetic analysis shown in Fig. 2**

| Species | OBP | BLAST accession number |
| --- | --- | --- |
|  | CfOBP1 | XP_011251750 |
|  | CfOBP2 | XP_011268026 |
|  | CfOBP4 | XP_011268025 |
|  | CfOBP5 | XP_025262643 |
|  | CfOBP6 | XP_025262640 |
| *Camponotus floridanus* | CfOBP7 | EFN68669 |
|  | CfOBP8 | XP_025269029 |
|  | CfOBP9 | XP_011254774 |
|  | CfOBP10 | XP_011253322 |
|  | CfOBP11 | XP_011253326 |
|  | CfOBP12 | EFN60245 |
|  | CfOBP13 | XP_025262844 |
|  | HsOBP1 | XP_011144088 |
|  | HsOBP2 | XP_011145645 |
|  | HsOBP3 | XP_011145650 |
|  | HsOBP4 | XP_011145651 |
|  | HsOBP5 | XP_011150623 |
|  | HsOBP6 | XP_011150624 |
|  | HsOBP7 | XP_011139543 |
|  | HsOBP8 | XP_011145654 |
|  | HsOBP9 | XP_019697001 |
|  | HsOBP10 | XP_011147602 |
|  | HsOBP11 | XP_011147605 |
|  | HsOBP12 | XP_011148183 |
|  | LhOBP1 | XP_012228745 |
|  | LhOBP2 | XP_012215197 |
|  | LhOBP3 | XP_012215200 |
|  | LhOBP4 | XP_012215201 |
|  | LhOBP5 | XP_012215202 |
|  | LhOBP6 | XP_012215203 |
|  | LhOBP7 | XP_012231124 |
|  | LhOBP8 | XP_012215198 |
|  | LhOBP9 | XP_012220368 |
|  | LhOBP10 | XP_012218066 |
|  | LhOBP11 | XP_012217929 |
|  | LhOBP12 | XP_012224274 |
|  | SiOBP1 | NP_001291522 |
|  | SiOBP2 | ADX94399 |
|  | SiOBP3 | XP_011157711 |
|  | SiOBP4 | XP_011157725 |
|  | SiOBP5 | XP_011156042 |
|  | SiOBP6 | XP_011165204 |
|  | SiOBP7 | XP_011167532 |
|  | SiOBP8 | XP_011170254 |
|  | SiOBP9 | XP_011173007 |
|  | SiOBP10 | XP_011171270 |
|  | SiOBP11 | XP_011171271 |
|  | SiOBP12 | ADX94408 |
|  | SiOBP13 | XP_011157738 |
|  | SiOBP14 | ADX94410 |
|  | SiOBP15 | ADX94411 |
|  | SiOBP16 | ADX94412 |

**Supplemental material**

**Amino acid sequences of the odorant binding proteins used for the phylogenetic analysis**

>LhOBP1

MHVSVILGAVLLQAIYVSAGPPDWVPPEMLEMVQGDKQRCMAEHGTSQALIDEVNDGKLTNDRSITCYMNCLLDAFSLVDEEGNLEAEMLISVIPEEFQEIGNKILNKCAVQAMVHGLIPMEKSINMTTSSNRSRNKRETKR

>LhOBP11

MARENLILICASLIVAQLVIVSTDDIDWSTMHDELRKLGAPIRKKCVAEVGVALETLDKVELGEFPTDDKKLACYFKCVMEKGGVLKKDGKINTKLMTKMLPQQVKHIGQEMLNECKTIEGANNCEIGFNFNKCMYQANPVAYFVI

>LhOBP9

IYFILHVRVRADIKRDCRRQTNVTWESLRQLKAGNIEQNDMKLKCYLRCFMMKSGILNKDNNIDVEKVLRYLPRNMQESSRRTLNRCKSISAQNACDKAFQIAMCYFKEHPEILKNAPII

>LhOBP7

MKAIVIILVVFVVAALGGLTDEQKARLKTYKESCISETGVDPVAVENAKRGEMAENDEKLACFANCLLQKIGFISADGDVNWEVIRAKVPSDVLQDQVDQLQNKCQGVDGSGCQKGAKLFKCFLKNKNFHLLS

>LhOBP5

MKYCKMCTGLLICVIMVAFVAVNQADAAMTMEQIQKTAMTMRNTCTTKSHADAGAVAAIQNGEFPDDNQLLKCYTLCVMKTMRTFKNGRVDDGMLTKQVDLMLPPDLSVPMKATAIKCAAEPPAGDDCATAYQFVKCSYQTDPNHFFFP

>LhOBP6

MRTVIVILFVSFVLAVNLRSAESRRMTFEEIKEALQPVKKHCIERVGTDIKLIDNANKGKFVPDRKLQCYYKCMLLNTKAMKDDKIIEQAFKNNVKLLLLEQFVEPVTKAIDHCRPIAMKPLEGCELAYELAKCYYEYDPSLVFYP

>LhOBP10

MRLLAITVGFLLQAWIVSCGTRPSFVSDQMIATAASVVNACQTQTGVATADIEAVRNGQWPETRQLKCYMYCLWEQFGLVDDKRELSLNGMLTFFQRIPAYRAEVEKAISECKGIGNYFAKGDNCEYAYTFNKCYAEQSPRTYYLF

>LhOBP12

MGVVSLADVKDVKKQLKAAIKSLQEDVVACISENSLVQNDWYDEEQIMTNLHVQSGNEQRTRRCGCTIACVLKRQNVINGSNINEIRMHDLIDNAVADSNQPSVRQLHKVVHKCVKEVKDITDECEKSFAIYICVAKAAHIAEGHEEHQPAEEESETKPEN

>LhOBP4

MIKSFALLVCLMATAVVANASTIADAALHGGSLSTEDLQQCYENANLAETDLITNVEIKNGSYKNPENEEKTRKNGCFTLCILQKRGIITESEIQKDKLYGKSAHAHLNPGTQAAIYATVHRCVEQVKTKPDMCDKSLDLLICLWKDFM

>LhOBP8

MKRAIFILYLFIALTTSVNDDFAERIAEELSISKTDMQICMNKTNVVPEDFMKLDQLMYDDVK

TVDINKSVLKVGCLFACLLQKKGMLLGPHINVEKIKEFVNTRMRSHTDSEGIAIGNRILDKCTDQVKSKTDECEVAIKILLCAATEINRLRNV

>LhOBP2

MKRIVLFVLALVAAAIANEEETVKHLAEKFETDVQTVEMCFQEADTNTAEIHAAQQTFMEMKSENDLDEETKKVIMKHGRFLACMLEKKDMMKDSKLVLDKILEMMDSEETKHGPSKEDITECVNEINEANEMNKEDRALELILCFMDDQEITAKNKK

>LhOBP3

MRKIAFLVLTLVAIAIADEEEDITDLAEEYETDEKTMRICLDETEINRDEIDAGWKKWAEIINNGNFNEEAKQSVAKLNRFIACLLEQKNMMKNSKLVVSKILQEMVDHNKDKKMPSEQIITECIIDLNKNDAMTKEDRVFGLLVCFVSNYGGKR

>SiOBP1

MHVSVILGVVLLQAIYVSAAPPDWIPPEMLEMVQGDKERCMAEHGTSQALIDEVNDGKLSNDRSITCYMNCLLDAFSLVDEDGNLEAEMLISVIPEEFQEIGNKILNKCAVQDGADKCEKIYNVAKCVQGTVPELWFMV

>CfOBP1

MHVSVILGMVFLQAIYVSAGPPDWIPPEMLEMVQADKERCMAEHGTSQALIDEVNDGKLSEDRSITCYMHCLLDSFSLVDEEGDLETEMLLSVIPEEFQEIGNKILTKCAKQDGADVCDKIFNVAKCVQGTVPELWFMV

>HsOBP1

MRTVYVSLILGVVLLQAICVSAGPPDWVPPEMLEMVQGDKERCMAEHGTSQDLIDQVNDGHLSDDRSITCYMNCLLDAFSLVDEEGDLEADMLISVIPEQYQEIGTKLLTKCAKQDGADPCEKIFKVAQCVQGTVPELWFMV

>SiOBP11

MARKSLILVCVSVVLTQLVVVSFSAKTDEIDWTTVHDDLRKLGATFRKKCLAETGVTIDKLEGAEMGQFPDDRKLACYFKCVMEKGGVMKKDGTINYKVLAKLLPQAYKQIGIDMMDECRDIEGSDSCEKGMKFHQCMYNANPVAFFVI

>CfOBP11

MATENLILICAFLIVTQLVIVHCNEANIDWTTVHDELRKLAGNLRKKCTGEIKGITDEMLEEAELGNFSEGDNKLACYFKCVMDKGGVMKKDGKINYKLLSKMMPAAYRHIGQEMLDECRNISGDDKCDVALNFNKCMYRANPVVSKKI

>HsOBP11

MARGNLVLICASLIVTQLIAVSSEEIDYTTIHDELRKLAGNLRKKCLGENNSITDEILGAAEQGDFENKGLACYFKCVMEKGGVMKKDGKINYKLLTKMLPPAYKQIGLGMIDECREIEGSDKCEIAFNFNLCMYNANPVAYFVI

>HsOBP9

MKAASILLVTLAGVLILLVRVRGDIKRDCRQQTKVSWDSLKQLKAGNVEQDDMRLKCYLKCFMMKSGILNKDNTIDLEKALRHLPRSMQESSRRILNRCKSIPADNACDKAFRIATCYVKEQPEILKSAAFV

>CfOBP9

MKTENMLFIVLLSVLFLLVRARADIKRDCRQQTNVSWASLKRLKAGNVEENDIKLKCYLRCFMMKSGILSENNNVDIEKAVRHLPRSMQESSREILNRCKSIPTENVCDKAFQIAKCYVKAQPEILKSVSFV

>SiOBP9

MKIINILLVILFLLLVGARADIKRECRQQTNVSWASLKQLKAGNIEQNDMKLKCYLKCFMVKIGILNEDSNVDVEKALRHLPRSMQESSKRILNQCKLIQAENACDRAFQIAICYVKAQPEILKNVSFI

>SiOBP7

MKAIIIVLAISFVAVLGQLTDEQKAKLRTYKESCINESGVDTTILENAKKGEVAESDEKLACFSTCLLKKIGIMNADEDINWEVARAKLPPGVPQEQADQIYNACKDITGTGCEKGGKVFKCFLDNKHFHLLS

>CfOBP7

MYVHKMLPRLLYYIFAEKNRLQMISKTYYLSISIVVVENAKNGQVAEGDEKLACFGNCILKKLGIINANGDIDWEVARSKVPPGISQEQIDHVYNKCQNVAGSGCEKGANLLKCFKENKNFAVLS

>HsOBP7

MKAIVVILAVSFVAVLGQLTEEQKAKLKDYKESCISETGVDRDVVKNAKEGVIDENNEKLACFATCLLKKTGVMKENGDIDIDVVRSKMPPGISQEDVDDLIQKCQNITGDGCWKGGALMKCIMENKLISLAKSHR

>SiOBP8

MHHSASTHAIAIFMSVIYILFVVVIITRFKETYLSENSPFTEKETEKLRGYRTLCMNKYDVDTTIIKKAKEMENIIDYIDERLVSYVICLYKNWGIINADGHIDWKVTLSMLPGVPQKVFNEIYSACNRTTGTDYERGYELFKCFLQNEVDLL

>SiOBP5

MCARLLICMITIALFAVHQTNAAMTMEQIEKTALNVRNTCTSKSHADPGAVAGIQNGVFPDDNKPLKCYTLCVMKTMRTFKNGRIDDGMMIKQVDLMLPAEMAGPLKAVATKCAAEPPTGDDCSTTYQFVKCSYSTDPDHFFFP

>HsOBP5

MASETRARTLICALVIALVAMQQANAAMTMEQIEKTAATLRNTCVSKVNANMDTVLAIQKGEFPDDRSLKCYTHCIMKTIRTYKNGRVDEGMMLKQLDLMMPADIAVLMKETTKICAAEPPTGDDCETTFNFVKCSYNNDPDHFFFP

>CfOBP5

MCIRVFIYIIMIALFAINQSDAVLTMEQIHKTAMTMRNSCVSKSHADAGAVEGLQTGQFQDDNQSLKCYTLCVMKAMRTFKSGRIDVGMMLKQMDLMMPVEMATPLKAVTTECAGHPPAGDDCETTYQFVKCCYQTDKDHFFFP

>CfOBP

MKGASVSFLISFVLIANLQNTESKKYSLDEIKVALKPVSDICIERVGVNPKIIEDANNGKLVPDRKLQCYYKCVMLMTKVMKNDKIVEKALQNIVELMLVEELQTIALGALKHCQSTLSKSMEGCELAYESVKCLLDYDSTFVL

>SiOBP6

MKETGTIFFISLVFMANLQNIESKRYTFEDIKVALEPLKKHCIDRVGKDQKVIDDANNFKIVPDWKTQCYYKCIMLNTKMMKNDKIVEKALINIAEHMLLEEYLPVVVKTIEQCHSTATKSMEGCALAYEYYKCLYDVNPTIAMYA

>HsOBP6

MKNVVLFVSLVLVTTLFQNAQSKRLTFGEIKETLLPVRKICMERVSIDSKMVDEANKGNFVPDRKLQCYFKCMMVMTKTMSKDDKVQRQSFTRTVQIMLEEKYVEPVLKAIEHCGPVAEQSLEGCQLAYEVTKCFYDYDASVRHACICKYRLNHLAHFMTIFSVCVTSESFQTSVAFAAHVLSVKTSLFVDPSAHLEGRNDYSFIVNAETISLFDRTKQHKNSQLFSIVL

>HsOBP10

MRLLAVAVGFLLQAWIVSCGTRPSFVSDQMIATAASVVNACQTQTGVATADIEAVRNGQWPETRQLKCYMYCLWEQFGLVDDKRELSLNGMLTFFQRIPAYRAEVEKAISECKGIGNYLAKGDNCEYAYTFNKCYAELSPRTYYLF

>CfOBP10

MRFLAVAMGFLFQAWIVSCGTRPSFVSDQMIATAASVVNACQTQTGVATADIEAVRNGQWPETRQLKCYMYCLWEQFGLVDDKRELSLNGMLTFFQRIPAYRAEVEKAISECKGIGKYLAKGDNCEYAYMFNKCYAELSPRTYYLF

>SiOBP10

MRLLTVALGFLLQAWIVYCGTKRPSFVSEQMIATAASVVNACQTQTGVATADIEAVRNGQWPETRQLKCYMYCLWEQFGLVDDKRELSLNGMLTFFQRIPAYRAEVEKAIGECKGLGNYLAKGDNCEYAYAFNKCYAQLSPRTYYLF

>SiOBP16

MKSLVLCACVLLFAFQLSSSTELKEKLKNEEKNIENILETCLNEQGLSRNDMYKEEELMTKVHTESVNAERTRKVGCFVACAMEKLNLMDEATIKETQIHAKINELFEGRDQGIAHKIARKCLKKARSITQKCEKCFSLYVCIAESVHKLQGHEEHVREETEEIEETEEQI

>CfOBP13

MKIIVLCVCVLGFASSNLIEDEIKRQFKSAVSREDNFETCMTENNVTFDDWYREEQIMTDVHKKPENEEK

TRNLGCTIACFLKKENLIEDSKIKEGKVHAKINKIYDGSRDEGKAHKIARDCMKEVKNITEECEKCFSLF

TCAVRAVHKSQKHEEHEGPEINENEETEQTI

>SiOBP12

MKHLVLCACVLLFTFQLSNSSELKQKIQEEVKIKQRIDFEACLSENGINESDLYGPTEIVSNVHTETANEEKTRKNGCFMACFLKKQNLMEGTNIKDEVIARLNEVVVTDDVEEKLRTIVRKCIKEKKDITQECDKCFPIYVCIIKAVNEERKCMQEENVRTEEEETGEPNKKK

>SiOBP13

MKHLVLCACVLIFALSNATEIQQEIQNEIKLRLDLEACLIENGLNNSGLYSMNEVSINVHTKPGNEERTRKNGCFMACVLKKQNLMEGTNIKEDEVIARLYELTRQDLKVILGKIVRKCLEEKRDITQECAKCFSIFECIIQTMDKFPREHEHEEIVTTE

>CfOBP12

MKIIVLCVCVLGFALSDLVGNKTIRQSAEEAEGALREKLETCMTENNVTFDAWSEAVQILINVHKKPENV

EKSRKLGCILACYLKKQDLPAILTKDFKMDDKLASDLTKLLQHLPD

>SiOBP14

MKGLILWVCVFIFASSSSSKLKEEKHINTNVRAIQDEIKPCLSEIGIAYEALYPPAEIIANVHTQPANKERTKNHGCFMACVLKKQNLIEGTNIKEAQVYSRLHEILDEELDGPGHQIIRKCMEEVRNMTQECEKGFSLYVCIKEAAAHEEEAKRQKNKKN

>SiOBP15

MKTFVLCACVFVLAVYFQSSNSSELNEQELRKIGISIRNDFNTCFSEIGITPADFVKPMEIVTNVHLQPANEERTNKHGCFIACVLKKQNLIEGTKIKEEQVYERLQLIFDENPGGPMHQIVQKCMEKVRNDAQECEKCFSVYVCTIKDMYEEEQRRKNERN

>HsOBP12

MKAVVFCACILVLYTFSSVEAIQNKLKHMLSEEDNDRPIDICLTQFNMTILDLYTEPEIMNEEYTKPENEEKNRKNGCLLECLLKKQGFMEGSDINETKIHTQMNTKFANDPMLGKMHTTVHKCVKEIKKNNITQECEKGFYLLTCMMKGMYKAKKHDEHESEHEHTT

>CfOBP4

MKLLALLVCLMAIAITYANTIADIALHGGLLSAADLQQCYENANLAEADLIINTEIKDGSYKNPENAEKAKKNGCFTLCILEKRGLIVDSEIQKNKLYGKSAHANLNQATQAKIYATVDRCTEQVKTVTDMCDKSLDLLTCLWKDFI

>SiOBP3

MKTLVFHIFIFALVAFASASRNSAKKIGSQYDHYQTCLTELGVTEDDLFSIGEVTSGQHKTKHEDTKLHRNGCVMQCLLEKAGLMTGADFDEEKMRENYIEEKGLQPGDQRIDFLNSCMEQTKDIEDKCDKSLIFIGCVLMNEVSLPASNEEA

>SiOBP4

MKTLVFHIFIFALIAFASASCNSAKKIGSHYDHYQTCLTELGVTEDELFSVGEVTNGQHKTKHEDTKQHKNGCIMQCVFEKLGLMIGADFDEEKMREYYIKEKGLQPGDQRIDFLSSCMEQTKNMEDKCDKSLGFIGCVLMNEVSLPASNEEA

>CfOBP8

MKQIIFVVFLFIAITMGLSDDVVERMAQSFSLPNIDVQICINKTVVKIEDLMKMDELVENSIQESSTDID

KSSSVLKIGCLLACLFQKKEMMSGAYINQIKLKEFLNTKMPRTDYETITKRDQILETCIDRVKSKTDECE

VMLKFTLCVILEAKDYVGL

>HsOBP3

MKRSLALLLIFVIPTIISADDFVEKLAEMLSLTQEDVQKCIEKTGTTQDDIMHFDQIVTDNLQTVDFDEK

AKRLGCFFSCLGQKMGIMTDGHLNVDKMKQIIRSKVKNPEKLAVGYRILDICNDQVRSKTNECEISIRFL

LCMADETERLRKNDMQRRYEDNNDNE

>HsOBP4

MKRPLVLFFTLVVLTIISAEDYVEKLAHSLSMTREDVQQCIGKTDATPEDIMHFDQIVLDNLQTVDFDEQ

ALRIGCFLTCLSQKSEIMYGASMNVEKLKLFVRSKAKEPNPDMIAIVYQTLDMCNDRVKSKTNECEVSFK

FLLCFIKEMRRLDENKTQRPYENTIDNE

>HsOBP8

MSEDYVGRLMNIAKLKREKVLECMNKTHVTIEDFEHFDRVLIDKLVVDFDNVVLKVGCLFSCIFQEKEVMTGAHIDIDKIKDMLRSKFRRDDTKQLDVRLQLLDTCSNEVKSNINECEVSLKFIICGLRETEKILNKDMDNTNDE

>SiOBP2

MEKVAFFVLALIAVVVANEIVEEMAKKFETDSATVQKCLDDTGITMEELGTSLKEWAELKDEDINEMTKQSLMKYVNFLACMMEKNEMMIDSKLVVDKIVESAQNDKDLLPPVPKEVLTECLTALNENSEISREDRVFGLMFCMMDGQTDKK

>CfOBP2

MKRIALLVLALATNAIADEDEMIQMEAVDFETGIQTIKDCLDGAETTIDELNLIRQKMLEIKEDEDIDEETKEYFLTYGRYIACILEKEDLIKDSKLVVDKIMKENEKDDTPLNKKDLEKCLNSLNEGDLNLEQRAFGLVLCFQNVKLASPNKR

>HsOBP2

MKRIAFLVFVFAASAIAEDAKELKEMAENFESDVQTVQLCLDEAETTESEMKAIKEKIDKLDTLDDNIDD

ETKKSLEKTNRFIACMLEKKEMMVDSKLNVDKILEAIEKDSKYHVKQDEVKECLNTLNNDKELDREMRAMGMMICLIKNEKDEDEKK

**Amino acid sequences of the chemosensory proteins used for the phylogenetic analysis**

>HsCSP1

MGKSAFCLAALAVLAAVVAEELYSDMFDHINPDDILPNDELRNQYYNCIMDTGPCVTDDQKYMKEHAAEAFATKCRKCTEVQKQNLEKVIGWYTENRPDEWTALMQKLMEDAKKMNISPV

>HsCSP10

MAWLVSIVAIIGIALVSVLTAEEEFYSDKYDDIDVKSILENDRLREQYRDCFMDKGSCTTADMKFYKEIVGDAVTTKCKRCTEIQKQNVDIITDWYTKNEPDKWREFVVKSLEDSKKKNGGQ

>HsCSP11

MARLIGIVAIIGIALVSILTAEEFYSDKYDDIDVKGILQNDRLRDQYYECFIDKGPCTTADMKFYKEIASEAMVTKCKKCNEKQKEHLNDIIEWYTQNKPDQWQIIVEKSLEDMKKKNSGQ

>HsCSP14

MTTRLIGVYLVIIFGLMCVYAEEELYSDKYDNIDIDGILNNDRLRNQHRRCYIGLAPCITADMKFYKKFIGEAIATKCRRCTEKQKQNLNKLADWYVTNKPEEWNEFIAKMIEAQREKNRGR

>HsCSP2

LVLGCALLAAAMPAEPDVEQTGRSRVSDEQLNMALSDKRYLARQLKCALGEAPCDPVGRRLKSLAPLVLRGSCPQCSNEETRQIKKVLSHIQRSFPKEWNRIVQQYAGVP

>HsCSP3

MKFALVCLLALVAVVYVSAKPQGYTTKYDNIDVEQILHNDRLLQRYVDCMLDKVGVRCPPEAIELKKVLSDALDTECNKCNDRQKEVAKKAIRFLIDNKPDIWKELKAKYDPEGKYVKKYEKLAEEEKIKF

>HsCSP4

MGYLAVALLTVCVLSSPVVLADNKGQYTTKFDNVDVDAIISSERLLNGYVGCLLERNPCTPDAAELKKNLPDALEHDCAGCSEAQKNAADKISHHLIDHKPEDWRLLEEKYDPTGAYRRRYLENKSKEGGRFD

>HsCSP5

MNKQQIIVALLSFSLGIIVFCEGKELSELLQDDKFIDSVKECILEKEKCDDMGFKVRNVLLPELISRDCESCSPSLKKDGQTLISNLKERFPTEWIIISHMYDQEIYDIPGYYTTPLMPPL

>HsCSP6

MKEFLLVSLVSLIALAVAAEKYPEKYDNVDVDRILQNNRVLTNYIRCLMDEGPCTAEGRELRKTLPDALSTGCSKCNDKQKATAENVINHLKMKRSKDWDRLISKYDPNGEYRERFERS

>HsCSP7

MQVVALLFLVVVCVLAEEKYTTKYDNVDVDSILASDRLLKNYVNCLLDKGSCTPDGKELKEHLPDALASDCSKCSEKQKRGSEKVIRFLVNKKPETWEDLKKKYDPTGQYTIKYQEDAKKQGLNV

>HsCSP8

MKLALVLFSFVFCNPVTGIEYYTATYDNIDVDAILNNERLFTQYMGCLLENGPCTADGRTLRRILPEAITTRCEKCNPRQKQIAKKISNHLKEKKPDIWIMLIEKIDPQEEDIAAFEEFLAQKEEEYLLR

>HsCSP9

MARLIGVYLVIIFGLMCIYAEELYSDKYDDIDVISILKNDRLRNQHRKCYLGVAPCTTADMRFYKDILGEAIVTQCRKCTEKQKQNLEIMTEWYITNKPDEWNKFVAKMIENLREKNKGQ

>SiCSP1

MDRLNFYLLAILAVLATIVAQETYSDMFDHINPDEILPNDELRNQYYNCFMDRGPCVTDDQKYFRQNIAEAFVTKCQKCTETQMKNYGKIVEWYTENRPDEWQAMVEKLLEEAKKLNITPA

>SiCSP10

MARLSSIALIIVAMNVLMCVFGEELELYPREIDDIDVLKILSDDAWRRRAEDCYFKRVPCAKEKQYLSDIFKDMLKTKCEKCTEKQKKLVKTATEWYEQNEPDTWKLILEDAHS

>SiCSP11

MARLSCIVTIIGITLMCVIAQEDLYTDKFDNVDVPGIIANDKLRNEYYGCFMGSSPCITADAKFLKEVFSDALNNNCKRCTEKQKEHMDYIVDWYTKNKPDEWQAIVVKSIEDLKKKNA

>SiCSP12

MAQLNRIALIFIAMSVLTCVLAEELWFYSGEFDDMDVLSILEAQAEQEVDCYMKRGPCTLEQQRIADSIREAIRTNCRRCTPKQKQQIQLITDWYKSRMPQNWELIVANVDL

>SiCSP13

MARLNRIALLVVATSVLMCILAEELELYPSELDDIDVAKILENDAERKGELNCYLKREPCAEEFNKYTEIFREAVRTNCKRCTEKQKEHLETITNWYKKNQPDNWELILENVNL

>SiCSP14

MARLSNIVLIIAVNVLICVLAKEELYSEQYDHLDVRGVLANNIQRKSYYNCFMGITPCTSEQKNLIFPDLFSEAYQTKCRKCTKKQIEHLNVISDWYTTHQPLKWLQLIQKMINDLRKKYANDH

>SiCSP15

MARLSCIITIIGIALMCVATQEDLYSDKFDGVDVPGIITNDRLRREYYNCFMGTSSCVTADAKFFKEIFFDALGSKCKRCTEKQKENMNFIVDWYTTNKPDEWQTLVAKSIEDLKKKNARK

>SiCSP16

MARLSCIVTVIGIALMCVAAQDLYSDKFDHIDVASIVTNDKLRNEYYSCIMDTSPCKTADAKFLKEIFAEALNNDCKKCTEKQKEHMKTIQDWYTTNKPDEWQAAVAKAEDLKKNAR

>SiCSP17

MARLSCIVTIIGIALMCVAAQEDLYSDKFDGVDVASIIVNDKLRNEYYGCFMETSPCITADAKFFKGVFADALNNKCKRCTEKQKEHMDYIVDWYTKNKPDEWQALVVKSIEDLKKKNARK

>SiCSP18

MARLSFIVTIIAVALACVLAEEELYSNRYDDIDIDRILENKKLRLQYYNCFMDTEPCRTADAKFFHEVISEAMQTQCRRCTEKQKVLLNRMADWYTQNAPEQWEAFIRKTLEDTLQKKG

>SiCSP19

MARLNYIALIVVAMSALMCVFAGDLGLYPSELDDLDVVALLADAAWRQQSDDCFLNKGPCSEEQKYLNDLFREAVRTDCERCTDKQRQIMNTITEWYEQNEADVWKIILEDARA

>SiCSP2

MLQILLVLLCALLAVAMATESSTDNVEGQQTGRSRVSDEQLNIALSDKRYLNRQLKCALGEAPCDPVGRRLKSLVPLVLRGSCPQCSPEEIRQIKKVLSHIQRSFPKEWNRIVQQYGAS

>SiCSP20

MTRLNSIALIIVAMNVLMCVLGEELELYPPELDELDVPQLLADDAWRGNIEDCYFKRAPCTEEQKYLEDKFRYALNTNCKRCTETRKKCMKTVTEWYEKNQPDTWKLVLENVDS

>SiCSP21

MARLSCIVTIIGIALMCVVAQEDLYSDKFDGIDVKSIITNNRLRNEYYDCFMGISPCVTADAKFFKDIFFDALGNKCKRCTEKQKEYMKIIQDWYTTNNPDKWQAAVAKSEDLKKKNARK

>SiCSP24

MARLNCIALFIVATSVLMCILAEELQPYPSEYDIYVPKILANDVVRQKAVDCYLKKGPCTEQEKLATDLFRDALKTNCKKCGEKQKEHVKILTEWFVKNQPDTWKLIIENVDS

>SiCSP3

MKLTVFCLLAVISVVYVYAEEKYTSKYDNIDIDQILQNDRLLKRYVDCFLEKPNVRCPAEALEAKAHIQEALDDECAKCSDHQKEMSKKVIRHLITNKRDMWNELKAKYDPDGKYAKKYEDEAKKEGVEI

>SiCSP4

MRHLVVTLITVYILSFSCVFAQEGTYTTKFDNVDVDAIISNDRLLNGYVGCLLDRNPCTPDAAELKKNLPDALEHDCAGCSETQKNAADKISHHLIDNKPDDWKLLEDKYDPTGTYRRRYLESRSKEGGSVD

>SiCSP6

MKGYFLVVLVSLVVLAVADEKYTRKYDDVNVDKILQNNRVLTNYIRCLMDEGPCTAEGRELRKTVPDALSSGCDKCNDKQKAMTEKVIDHLKTKRSRDWDRLVAKYDPNGEYKKRYEKS

>SiCSP7

VLALFLLVVAIALAEEKYSTKYDNIDLDTILKSDRLLKNYVNCLLDKGNCTPDGKELRETLPDALMTECKKCSEKQKEGTEKVIRYLVNKQKPETWEQLKKKYDPNGQYTAKYLDEAHKQGINV

>SiCSP8

MSLTFVLLFSLAFSGLVSGIEYFSDNIDVDAIINSDRLLNQYVNCILDKGPCTADGRSLKFLPDAIATTCEKCSEKQKQTARKIIKYLKEHKPNIWAEFLERYDPDEEHVAFYKEFLAQG

>SiCSP9

MARLNCIALLIVATSVLMCVLAEDLHSELDDLDIPKILANDAERQGVIDCILENASCTELETKAAAAIKDALKTNCQACGDKRKENMKIITDWFNQNQPDTWTLVVAKVNS

>CjapCSP1

MDKSSLCLLALGVLAAVIAEEMYSDMFDHINPDDILPNDELRNQYYNCFMDTGPCVTEDQKYFKEHAAEAFATKCRKCTEVQKKNVEKIVVWYTENRPQEWQAMVQKLMDDAKKLNIPFTR

>CjapCSP2

MALTIKFLILVCALFTATMAAESDNSEGQQSGRSRVSDEQLNIALSDKRYLTRQLKCALGEAPCDPVGRRLKSLVPLVLRGSCPQCSPEETRQIKKVLSHIQRSFPKEWSRIVQQYAGVS

>CjapCSP3

MKFALVCLFAISTIVCVYGRPQDHYTDKFDNIDVDQILNNDRLLKRYVDCLLERSHVKCPSEALELKKVLADAMATDCAKCTDRQKEIARKALDFLIINKTDMWNDLKSKYDPEEKYAKKYEDRALKKEN

>CjapCSP4

MKHLVVALITALSFSVVLAEDVQYTTKYDNIDVDAVINSERLLNGYVGCLLDRTPCTPDAAELKKNLPDALEHDCAGCSEMQKNAADKISHHLIDNKPDDWRLLEDKYDPTGAYRRRYLENKSHEGGRLD

>CjapCSP5

MNKQIIILIIIGSGLAVFCQAQDISSYLTDKRFIDKELHCLLETGDCDGFGKQIKRVLPVVLKDKCRRCTPQQKANLHKLIQFLQSRYPTQWHTIEEMYSSPTFQ

>CjapCSP6

MKKYLLISLASLMILVVATEKYTGKYDDVDVDKILQNNRVLNNYIRCLLDEGPCTAEGRELRKTLPDALSSSCSKCNDKQKATAEKVINHLKTKRSKDWDRLIAKYDPRGEYKKRYEQL

>CjapCSP7

MKVLALLLIAVACALADDKYTTKFDNIDVDAILKSDRLLKNYVNCLLDKGNCTPDGKELKEHLPDALETECSKCSEKQRTGTEKVIRFLVNKKPETWEQLKKKYDPNGEYSRRYEDEAEKRNIKA

>CjapCSP8

MKLPFLLLLSSFVFCGLVSGTENYTDIHDNVDIDAILNSDRLLKQYMDCILEKGSCTADARSLKRILPEAVATICEKCNLKQRQGARKIGNHLKKYKPELWTIFLEKYDPNKEYIENFEQFLAQVEE

>CjapCSP9

MTKLVSCTFACLVMTLAVLIAHAEDEKYSSKYDHIDINEVLANSRLRNQYVRCLINISPCTTGSARFLKDIQGEAFVTKCKKCTDKQIYILNAITDWFTKNEPETWNRMVQVAVEEAKRKNA

>CjapCSP10

MARPSYIVAIVVIALTCVLAEELYSSRFDDVDVRAIFNNAKLRNQYYNCFMDLSPCKTADQRFFKGIFSEALQSGCKRCTEKQKENLEIVLDWYTINDPIKLQTFIAKSIEDLRKKNSES

>CjapCSP12

MARLICTIAIIGIALMCVLAEEEKYEDKYDDIDVHEVLENVKLREQYYKCFMATGPCVTADQKFFSKIVSEAFQTKCKLCTEKQKYMLDEISEWYTKNDPEKWNAFIAKTLEDMKKKAKE

>CjapCSP13

MARLNCIIILISIASCVLAEELYSDQYDHIDVNNILNNDKLRDQYFNCYMETEPCLTAEAKFYRDIASEALQTKCKRCTEKQKEIIDAVVDWYTQNKPDKWQKIVEKSLEDMKKKNAGQ

>LhCSP4

MRHLIVALLTVCALSSTAVFAEEAPYTTKFDNVDVDSIINNERLLNGYVGCLLDRNPCTPDAAELKKNLPDALEHDCAGCSEAQKNAADKISHHLIDNKPDDWKLLEDKYDPTGAYRRRYLENKSREGGRFD

>LhCSP3

MKLALVCLLAIATIVYVSARPDGYTNRYDNIDVDQILNNDRLLKRYTDCLLEKANARCPPEAIELKKVLNEALETECAKCSEHQRQIVRKVIRFLVENKRDLWNELKAKYDPEGKYVNKYEDMATREGVQL

>LhCSP2

MALAIKILVLVCALLATTMAAESGSDGQQSGRSRVSDEQLNLALSDKRYLTRQLKCALGEAPCDPVGRRLKSLVPLVLRGSCPQCSSEETRQIKKVLSHIQRSFPKEWNRIVQQYVGVS

>LhCSP10

MIRLSYIVLIGIALACVVAEEELYSNQYDDIDIKAIFENEKLRQQYYNCFMEIAPCKTAPLKFYKEIFPEAFQTQCKKCTEKQKKHMEYIIDWYTTNKPNDWNALVTKIVQDLTKKSTQ

>LhCSP11

MVRLSCIVIISIALACVLAQEELYSAQYDNIDIVHLFENAEARQCVYNCFMDVGPCDSQSQFGNLKFFKGIFPEALHDGCKKCTEKQKEYLKYITDWYTTNEPDAWNALLTKIQNDFVKKDVQ

>LhCSP1

MGKSSLCLLAFLAVLAIGAAEDLYADTYDHIEPMEILNNDELRNQYYNCVMNTGPCMSDEQRFLKEHVAEAMATRCRRCTERQKDGLEKVVVWYTENRPEEWSALVVHLIEEAKKQNITPVSGGFI

>LhCSP9

MVQLSCIVFIGIALVFVFAEELYTSQYDYIDVDIILANNKLRNQYYKCFMETAPCKTADMKFFREVFSESLQTQCKKCTEKQKIILEHIIEWYTENRPDEWNVLIRKHLRDLAKKNAQ

>LhCSP12

MARLSYIVAIISIALTYVVAQELYTDKYDDVDVIQILENEKLRDQYYKCFMEQGPCLTGDAKFLREILSEAFQTKCKKCTEKQKVMLDQIVDWYTTNAPDEWRTIVEKTVEDMKKMNANK

>LhCSP7

MKILVLFLFVVACVLAEDKYTTKYDNVDLDTILESDRLLKNYINCLLEKGNCTPDGKELKAHLPDALTTDCSKCSEKQKKGTEKVIRYLVNKKPESWEQLKKKYDPTGQYSAKYVDEAKKQGINV

>LhCSP6

MMKGYFLITLALLITLVTAAEKYPEKYDNVDVDAILGNNRVLTNYIRCMMDEGPCTAEGRELRKTLPDALSSDCSKCNDKQKTTAGKVINHLKTKRTKDWDRLISKYDPRGEYKRRYEQ

>LhCSP8

MRLTLVLLLSFLASGLATGMESYPDTYENVNLDVILNNERLFNQYMDCVLDKAPCTADGHYLKRILPEAVATTCEKCNVKQRQMARKIGNYLKNSKPKTWAAFLEKYDPNKKYIATFEQFLVQIEK

>LhCSP14

MARLSYIVMVISISLMCVFAQEQYDNKYDDIDAMVILEDSKQRNEWYNCFKKTGPCLTPEAAFFGEILPESYQTQCQKCTDKQKLLLETITEWYTKNEPENWHSLVANIEVVHN

>LhCSP13

MVRLRSIVAIIIFALICVFAEDGYDTKYDDIDVNGILENNKLREQYYKCFMDQAPCMTADSKFFKEIVGEAYHTNCKKCNERQNIMLDTMVKWYKQNQPDKFKAFEEKTGREFQKKA
